# HIV knowledge and risky sexual behavior among men in Rwanda

**DOI:** 10.11604/pamj.2015.22.380.6661

**Published:** 2015-12-17

**Authors:** Etienne Rugigana, Francine Birungi, Manassé Nzayirambaho

**Affiliations:** 1 School of Public Health, College of Medicine and Health Sciences, University of Rwanda

**Keywords:** HIV, sexual behavior, Rwanda

## Abstract

**Introduction:**

New infections of Human Immunodeficiency Virus (HIV) remain a big problem in many countries. Different interventions have been implemented to improve the general knowledge of HIV, with the hypothesis that increasing HIV knowledge will reduce risky sexual behavior (RSB). However, HIV knowledge may not necessarily reduce RSB. This study explores HIV knowledge and its effect on RSB.

**Methods:**

The study used data from the 2005 and 2010 Rwanda Demographic and Health Surveys to analyze the association between HIV risk factors and two types of RSB (having two or more partners in the past 12 months; and among those with two or more partners, not using a condom at last sex) and the association between HIV knowledge and those RSB. Multivariate logistic regression was used to determine predictors of RSB.

**Results:**

Among 2,773 men in 2005 and 3,772 men in 2010, 5% and 7% respectively reported having two or more sexual partners. Among them, 93% in 2005 and 74% in 2010 did not use a condom at the last sex. Between 2005 and 2010, knowledge of the protective effect of having just one uninfected faithful partner, and basic knowledge of HIV decreased. Knowledge of the protective effect of using condoms increased from 90% to 94%. However, HIV knowledge was not associated with either type of RSB.

**Conclusion:**

In setting up policies and strategies related to HIV prevention, policymakers should consider that focusing on HIV knowledge is not sufficient in itself.

## Introduction

In Rwanda as in many other countries, different interventions have been implemented to fight against new infections of the Human Immunodeficiency Virus (HIV). Generally, one of the main objectives of such interventions is to improve general knowledge of HIV. Around the world, local and international efforts have led to successful HIV prevention programs in terms of improving HIV knowledge and awareness. However, HIV transmission is not decreasing as rapidly as desired, especially when considering the large amount of money and many other efforts spent on its prevention [1]. It is therefore important to understand the relationship between HIV knowledge and risky sexual behavior (RSB), and the different components of HIV knowledge that are positively associated with the reduction of RSB.

Since 1987, the government of Rwanda has been making substantial efforts in the fight against HIV/AIDS. Activities related to HIV/AIDS prevention and treatment has been carried out through different structures such as the National Program for the Fight against AIDS (PNLS), the National AIDS Commission (CNLS), the Treatment and Research AIDS Centre Plus (TRAC Plus), and the district AIDS committees (CDLS). Different strategies used in the fight against AIDS are also found in the Rwandan National AIDS strategic Plan. These include Information, Education, and Communication (IEC) strategies, which target mainly sexually active men age 15-59 and women age 15-49 [2, 3]. These efforts have resulted in the achievement of two objectives: first, increased awareness of the existence of HIV as well as its transmission. Data indicate that the proportion of men who have heard of HIV/AIDS was virtually universal, both in 2005 and 2010 [4]. The second objective is increased awareness of ways to prevent HIV infection. Under this objective, information related to the “ABC” strategy (abstinence, being faithful, using a condom) has been widely disseminated. Condoms have also been distributed free of charge in different strategic places or sold at very low cost, from the cities to the last small shop in villages. Despite a satisfactory situation in terms of knowledge of HIV prevention and availability of condoms, many people still engage in RSB. RSB can be defined in different ways. The U.S. Centers for Disease Control and Prevention (CDC) defines RSB as behavior that increases one's risk of contracting sexually transmitted infections and experiencing unintended pregnancies. Risky behaviors are measured through a number of elements that include having sex at an early age, having multiple sexual partners, having sex while under the influence of alcohol or drugs, and having unprotected sex [5]. Many authors include in their definitions of RSB two main elements: 1) having sexual intercourse with a casual acquaintance without using a condom; 2) having multiple sexual partners [6].

This study is about HIV-related RSB. We focus on two types of RSB among men that have been identified by international experts as indicators of risky behavior: multiple sexual partners (having two or more sexual partners in the past 12 months) and unprotected sex (not using a condom at the last sex for someone who had two or more sexual partners in the past 12 months). These two indicators-P8.11.N and P8.12.N, respectively, in the President's Emergency Plan for AIDS Relief (PEPFAR) are useful measures of risk behavior in the population [7]. Different studies have reported low levels of condom use regardless of the infection risks [8–10], while some others did not find significantly higher levels of condom use among populations with higher prevalence of HIV [11]. A study conducted in Ethiopia among factory workers with a high HIV prevalence also showed low condom use despite widespread of knowledge of condoms [12]. Similarly, an increase of some types of RSB was observed in Uganda over the period of 2001-2005, where a higher percentage of males age 15-49 in 2005 reported having multiple sexual partners and having sex with non-spousal partners compared with 2001 (25% versus 29% and 28% versus 37%, respectively) [13]. Such a trend in RSB is notable given the high level of HIV prevention knowledge and known reduced risk of contracting HIV in the case of consistent condom use [14, 15]. This is why the question arises about the effect of HIV knowledge on RSB.

The literature gives mixed results about the effect of HIV knowledge on RSB. For example, studies in Zambia, Uganda, and Kenya show a positive effect of knowledge of HIV prevention methods on protective sexual behavior [16, 17]. However, other studies reveal little impact on condom use of having good knowledge of its protective benefits [18, 19]. Empirical theories about the link between behavior and knowledge suggest that self-perceived vulnerability to HIV is probably the main factor underlying RSB, more so than knowledge [20]. The basis of HIV perceived vulnerability is people's beliefs about their own behavior as well as the possibility of getting HIV from this behavior [21]. Perceived vulnerability is an important component of the Health Belief Model. Specifically for HIV, it has been proposed that in the general population there is a negative association between RSB and HIV self-perceived vulnerability [20]. That is, when people perceive that they are less vulnerable to HIV, they would be more likely to engage in RSB, independent of their knowledge about HIV.

In addition to HIV knowledge, some studies have also explored other factors correlated with RSB. According to the literature of developed countries, associations between RSB and socio-demographic factors are fairly robust [22]. In other studies, some individual characteristics such as age, religion, caste, wealth status, education, occupation, and knowledge about sexually transmitted infections and HIV were associated with increased RSB [23–26]. Other factors associated with higher RSB are smoking and alcohol [13–15], as well as witnessing violence among parents [15]. Recent studies also have reported an association between male circumcision and RSB [27–29]. To sum up, many strategies aiming at reducing RSB assume that increasing HIV knowledge and making condoms available will increase safer sexual behavior. However, it is not always the case. The present study assesses this relationship between HIV knowledge and RSB in the context of Rwanda, where programmatic efforts in HIV prevention measures have been scaled up between 2005 and 2010.

Research Questions: In Rwanda, HIV new infections are estimated to be 6,000 per year (MOH (Rwanda) and Rwanda Biomedical Center (RBC) 2014). HIV prevalence is 3% in the general population, and 2.2% in men versus 3.7% in women. This prevalence is highest among women in urban areas, at 8.7%. It was also found that HIV prevalence is highest among those who are widowed, divorced, or separated, and those who are in polygamous unions [30]. Even though men's HIV prevalence in Rwanda is relatively low compared with some other African countries, Rwanda ranks 13^th^ among 27 sub-Saharan African countries [31], unchanged since 2005. Thus HIV still constitutes a big burden on the Rwanda Health System. Many strategies have been developed and implemented at the national level to reduce the incidence of HIV in the general population of Rwanda. These strategies include mass media communication, community events and outreach, and campaigns. Young people have been reached through school-based sexual health and AIDS education, anti-AIDS clubs, and outreach work for out-of-school youth. One of the main objectives has been to provide the entire population with comprehensive knowledge of HIV/AIDS [2].

However, very few population-based studies describe the results of these interventions in terms of status of HIV knowledge and its effect on RSB. Thus, the aim of this study is to fill in this gap, exploring HIV knowledge and its effect on RSB among men, based on data from the 2005 and 2010 Rwanda Demographic and Health Surveys (RDHS). Specifically, this study tries to answer the following questions: Are there differences in HIV knowledge between the 2005 and 2010 surveys? What are the differences in RSB between the 2005 and 2010 surveys? What is the association between HIV knowledge and RSB of men in 2005 and 2010?

The answer to these questions can help to design more focused and strategic interventions. This is particularly necessary not only for Rwanda but also for other comparable countries where governments and partners continue to implement and scale up HIV prevention interventions. It is important to implement HIV prevention programs based on strong evidence. This implies better understanding of the HIV dynamics and determinants that drive the transmission of new infections, and thus selecting and focusing on only those interventions that effectively and efficiently contribute to preventing new infections.

**Figure 1:**
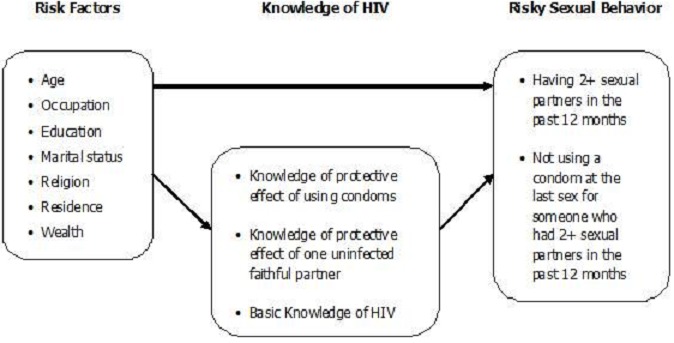
Factors model for predicting risky sexual behavior among men

Figure 1 shows the assumed factors in predicting engagement of men in RSB. Background characteristics (age, occupation, education, marital status, religion, residence, and wealth) constitute the risk factors of engaging in RSB (having two or more sexual partners in the past 12 months, or not using a condom at the last sex for someone who had two or more sexual partners in the past 12 months). The framework postulates also that knowledge of HIV can modify the effect of risk factors on RSB, which means that men who have the above-mentioned risk factors should engage in fewer risky behaviors if at the same time they have knowledge of HIV.

## Methods

**Figure 2:**
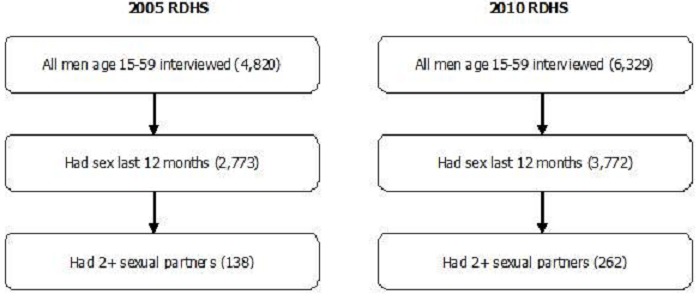
Derivation of sample

Data from the 2005 and 2010 Rwanda Demographic and Health Surveys (RDHS) [4, 32] have been used to analyze, on one hand, the association between risk factors and RSB, and on the other hand, the association between knowledge of HIV and RSB. The National Institute of Statistics of Rwanda and the Ministry of Health implemented the two RDHS surveys. ICF International provided technical assistance for the surveys through the USAID-funded MEASURE DHS program. The two RDHS samples were nationally representative, household-based surveys, designed to provide population and health indicator estimates at the national, urban-rural, and district levels. The sampling method used was stratified two-stage cluster sampling. The first stage consisted of selecting clusters (enumeration areas) from each stratum (district). The second stage was a systematic sampling of households from each cluster selected. Then selected households were visited and interviewed. The eligible men's response rate was 97.2% and 98.7% in 2005 and 2010 respectively. Eligible men age 15-59 were interviewed in half of the households. However, during analysis, only men who said that they had sexual intercourse during the last 12 months were considered, and this led our study population to 2 773 men in 2005 and 3 772 men in 2010 (Figure 2). The two surveys were conducted from February to July (2005 RDH) and from September 2010 to March 2011 [4].

### Key Variables and Measurements

During the interview, the following information on RSB was collected in the Men's Questionnaire: sexual activities in the 12 months preceding the survey, the number of sexual partners in the past 12 months (and the relationship with each partner for up to three partners), and condom use in the last sexual intercourse with each partner (for up to three partners). In total, 4,820 men in 2005 and 6,329 men in 2010 were interviewed. Among them, 2,773 men in 2005 and 3,772 men in 2010 had sexual intercourse during the last 12 months. Among those who had sexual intercourse, 138 men in 2005 and 262 in 2010 had two or more sexual partners (Figure 2). In this study, RSB was defined as having two or more sexual partners in the past 12 months, or not using condom at the last sex among individuals who had two or more sexual partners in the past 12 months. In fact, this is a metric of inconsistent condom use. The issue is that when a man has multiple partners and does not use condom consistently, he puts both partners at the risk of HIV infection, even if the most recent partner is a faithful spouse. HIV knowledge was defined as knowing that consistent use of condom during sexual intercourse and having just one uninfected faithful partner can reduce the chance of getting the AIDS virus, as well as having “basic knowledge” about HIV (knowing that a healthy-looking person can have the AIDS virus, and rejecting the two following most common local misconceptions about AIDS transmission or prevention: HIV transmission by mosquito bites and sharing food).

### Statistical Analysis

Analysis was performed using Stata 11 (Stata Corporation, College Station, Texas) to find the prevalence of RSB according to background characteristics (age, occupation, education, marital status, religion, residence, and wealth). Bivariate analysis was carried out and t-test was used to assess any significant change during the five-year study period. Also, logistic regression was done to determine predictors of RSB. Two binary outcomes variables were: “having two or more sexual partners in the past 12 months” and “not using condom at the last sex for someone who had two or more sexual partners in the past 12 months”. The following variables were tested in the model: knowledge of HIV and background characteristics. Chi-square test at the level of 5% was used to determine the statistical significance while associations were measured using odds ratios (OR) with 95% confidence intervals (CI). To account for the survey design and non-response, all analyses applied complex sampling weights.

## Results

A total of 2,773 men in 2005 and 3,772 men in 2010 reported that they had sex during the last 12 months preceding the survey. Among them, 138 in 2005 and 262 in 2010 declared that they had two or more sexual partners.

### Sample Characteristics

As Table 1 shows, the age composition of sexually active men remained fairly stable between 2005 and 2010, with men age 15-24 comprising the largest share of that group in both years (43% and 41%, respectively). Among sexually active men, however, those with two or more partners tended to be age 25 or older, with 32% and 40% being in the 25-34 age group in 2005 and 2010, respectively. The majority of men who had two or more sexual partners had primary education level (75% in 2005 and 69% in 2010), were located in rural areas, and were married (83% in 2005 and 73% in 2010). Among sexually active men, wealth index status remained stable between 2005 and 2010, with the middle, richer, and richest quintiles comprising the largest share. However, among sexually active men, those who had two or more partners tended to be in poorer (29%) and middle (24%) quintiles in 2005, compared with middle (25%) and richest (26%) in 2010. The occupation profile of sexually active men varied from 2005 to 2010. Men who reported not working decreased substantially, from 36% in 2005 to just 1% in 2010, while jobs related to professionals/clerical/sales/services increased from 8% in 2005 to 43% in 2010. These changes are associated with the governance between the two surveys, especially the “Economic Development and Poverty Reduction Strategy” (EDPRS I) established in 2008. The EDPRS focused on job creation and the reduction of underemployment by making micro loans through microfinance cooperatives across the country. Table 1 also shows that the majority of men with two or more sexual partners have the following main background characteristics: married, age 25-34, agriculture occupation, and rural location.

**Table 1:** Sample characteristics

	Among men who had sex last year		Among men who had 2+ partners last year	
	2005 (n= 2,773)	2010(n= 3,772)	2005 (n= 138)	2010(n= 262)
**Age (Years)**				
15-24	42.5	41.2	11.0	16.6
25-34	23.7	27.6	32	40.2
35-44	17.5	14.5	28.2	18.5
45-54	13.2	12.6	24.4	18.5
55-59	3.1	4.1	4.4	6.2
**Education level**				
No Education	22.9	16.6	16.0	13.0
Primary	64.8	68.8	74.7	69.4
Secondary	12.3	14.6	9.3	17.6
**Current Marital status**				
Not married	10.8	13.5	17.3	26.9
Currently married	89.2	86.5	82.7	73.1
**Occupation**				
Not working	35.5	1.1	27.7	0.2
Prof/cleric/sales/serv	8.4	43.3	10.3	15.7
Agriculture	42.1	66.7	43.3	58.0
Manual	14	20.7	18.7	26.1
**Religion**				
Catholic	51.5	47.8	42.2	46.2
Protestant	31.8	35	35.4	35.3
Adventist	12.1	12.6	14.6	12.8
Other	4.6	4.6	7.8	5.7
**Type of place of Residence**				
Urban	16.2	15.9	15.7	21.3
Rural	83.8	84.1	84.3	78.7
**Wealth**				
Poorest	19	15.4	12.1	12.7
Poorer	19.1	17.8	29	16.6
Middle	20.7	21.5	24.4	25.1
Richer	21.3	21.8	16.7	19.8
Richest	20	23.5	17.8	25.8
**Circumcision**				
No	89.8	86.3	87.7	81.9
Yes	10.2	13.7	12.3	18.1

### Trends in HIV Knowledge among Men

From 2005 to 2010, among all men who had sex in the year preceding the survey, basic knowledge of HIV appeared to decrease, while among men who had two or more partners in the year preceding the survey, it appeared to increase. However, neither change was statistically significant. The trends in the two other types of knowledge are similar among all men who had recent sex and among men with two or more partners. In both groups, knowledge of the protective effect of having only one uninfected faithful partner decreased, while knowledge of the protective effect of using condoms increased. The decrease in knowledge of the protective effect of one uninfected faithful partner and the increase in the knowledge of the protective effect of using condoms were only statistically significant among all men who had sex in the year preceding the survey (Table 2).

**Table 2:** HIV-Related knowledge among study population

	Among those who had sex last year				Among those who had 2+ partners last year			
	2005 (n = 2,773)		2010 (n= 3,772)		2005 (n= 138)		2010 (n = 262)	
	%	95% CI	%	95% CI	%	95% CI	%	95% CI
Basic knowledge of HIV	71.0	68.9-73.0	67.7	65.-69.5	65.3	56.6-73.1	68.2	61.7-74.1
Knowledge of protective effect of one uninfected faithful partner	89.8	88.1-91.4	82.0	80.2-83.6	89.2	82.2-93.7	83.9	78.7-88.1
Knowledge of protective effect of using condoms	89.8	88.2-91.2	94.0	93.1-94.6	92.3	86.6-95.7	96.9	92.3-98.8

### Risky Sexual Behavior by HIV Knowledge

**Figure 3:**
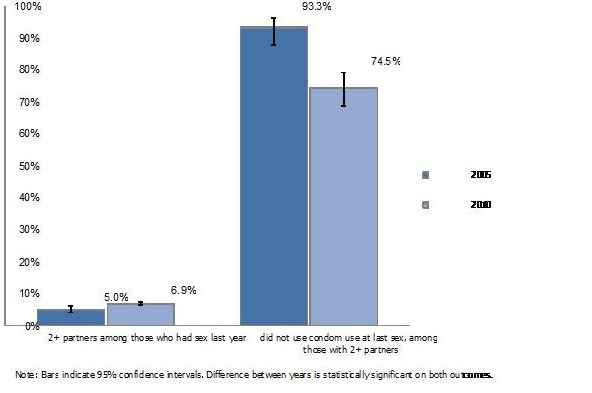
Percentage of men who had two types of risky sexual behavior

In 2010, more men reported that they had two or more sexual partners compared with 2005 (7% versus 5%). However, among those who had two or more sexual partners, the percentage using a condom at last sex was higher in 2010 than 2005, at 93% versus 75%. The association is statistically significant (Figure 3). Table 3 shows that, among men with basic knowledge of HIV, knowledge of protective effect of one uninfected faithful partner, and knowledge of the protective effect of using condoms, a significantly higher proportion had two or more partners in 2010 compared with 2005. In 2010, among all knowledge groups, a statistically significantly lower proportion of men with two or more partners reported not using a condom at the last sex than in 2005, meaning that risky sexual behavior declined between the two surveys (Table 3).

**Table 3:** Risky sexual behavior by knowledge of HIV, 2005 and 2010

	Men who had 2+ sexual partners last year				Men who did not use a condom at the last sex among those who had 2+ sexual partners			
	2005 (n = 2,773)		2010 (n= 3,772)		2005 (n= 138)		2010 (n = 262)	
	%	95% CI	%	95% CI	%	95% CI	%	95% CI
Basic knowledge of HIV	4.6	3.7-5.7	7.0	6.0-8.0	95.1	88.5-98.0	71.3	64.5-77.2
Knowledge of protective effect of one uninfected faithful partner	4.9	4.1-6.0	7.1	6.1-8.1	93.8	87.6-97.0	75.1	69.0-80.4
Knowledge of protective effect of using condoms	5.1	4.2-6.2	7.2	6.3-8.1	92.7	86.8-96.1	74.0	68.4-78.9

### Association between HIV Knowledge and Risky Sexual Behavior

The study explored the association between HIV knowledge and risky sexual behavior using bivariate analysis and logistic regression. The model used is a pooled model of association between HIV knowledge and risky behavior. Each type of HIV knowledge (basic knowledge, knowledge of the protective effect of one uninfected faithful partner, and knowledge of the protective effect of using condoms) was regressed separately with each risky sexual behavior (having two or more sexual partners; not using a condom at the last high risk sex) in the unadjusted models. Then, HIV knowledge variables and background variables were regressed together with a set of control factors in the adjusted models. In unadjusted models, the only HIV knowledge variable associated with any risky sexual behavior outcome was knowledge of the protective effect of using condoms. Men who had this knowledge had higher odds of having two or more partners in the past year; interestingly, this knowledge was not significantly associated with condom use.

The year of the survey was not statistically significant in the adjusted model of having two or more partners, but it was statistically significant in the adjusted model of condom use, indicating that men with two or more partners in 2010 were significantly less likely to report not using a condom at last sex than men in 2005, even after adjusting for knowledge and characteristics. The odds of having two or more sexual partners are greater among men in the poorer and middle wealth quintiles, while married men are less likely to have two or more sexual partners compared with unmarried men. However, even after adjusting for other factors, married men have 37 times the odds of not using a condom compared with unmarried men, when they have two or more sexual partners. Professional occupations and manual workers have increased odds of having multiple sexual partners compared with men who are not working, but among those with more than two partners, men who work in professional occupations and manual labor have significantly lower odds of not using a condom. Rural-urban residence is unrelated to either type of risky behavior in the adjusted models.

## Discussion

This study explores HIV knowledge and risky sexual behavior. Using the 2005 and 2010 RDHS, we found a number of important results. First, among sexually active men, the findings show that knowledge of the protective effect of using condoms has significantly increased, comparing 2010 with 2005, and this is a very encouraging result. However, basic knowledge of HIV (knowing that a healthy-looking person can have the AIDS virus, knowing that HIV is not transmitted by mosquito bites or sharing food) has not significantly changed, and knowledge of the protective effect of having one uninfected faithful partner has decreased between 2005 and 2010, both among men who had sex in last year preceding the survey and men who had multiple partners. This result suggests the need for more HIV education particularly on the issues discussed above. Unfortunately, for the moment it is not possible to explain this decrease of HIV knowledge through HIV programmatic activities. In fact, it seems that there is no significant change in the implementation of HIV prevention activities since 2005 that could explain this decrease.

Our findings also show that in 2010 the percentage of men who used a condom at last sex significantly increased compared with 2005. In their study on HIV-related knowledge and behaviors in Rwanda, Rathavuth, Hong and colleagues also found a considerable increase in condom use during last sex and during last non-spousal sex [3]. This result is encouraging, and it may be the positive effect of HIV/AIDS prevention interventions that Rwanda has been implementing. For example, the observed increase in condom use could be linked to the significant increase of knowledge of the protective effect of using condoms (from 90% in 2005 to 94% in 2010).

In an unadjusted logistic regression model, sexually active men with knowledge of the protective effect of using condoms had 1.8 times higher odds of having two or more partners in the past year, a finding which may suggest some degree of reverse causation: men who engage in risky behaviors are more knowledgeable about the means of risk reduction. Otherwise the logistic regression analysis did not find any relationship between HIV knowledge and the two types of risky sexual behavior studied. Although a bit surprising, this finding corroborates the results of other studies [18, 19]. In their study on HIV/AIDS information and risky behavior in Botswana [33] also concluded, “it may be overly optimistic to hope for reductions in risky behavior through the channel of HIV-information provision alone.” Therefore, this is a very important subject, particularly for policymakers who decide on strategies, interventions, and activities for HIV prevention. It is necessary not to focus only on AIDS knowledge as a means to reduce HIV transmission. Further studies could provide more information on other elements that probably influence safer sexual behavior and that therefore could be a focus for designing HIV prevention programs.

Our study has some limitations that should be considered when interpreting its results. Few men reported having two or more sexual partners (only 5% in 2005 and 7% in 2010). On one hand, this could be an under-reporting of sexual behavior. In fact, when men are interviewed in close quarters with other family members, they may not be willing to disclose their sexual behavior. On the other hand, the small sample size of men who had two or more sexual partners could be a reason for lack of significant association found between HIV knowledge and condom use. It is also possible to have recall bias in condom use among men who have not recently had sex.

## Conclusion

The findings suggest that, while knowledge of the protective effect of condoms has increased, basic HIV knowledge has declined slightly among Rwandan men, and this should draw attention and lead to take appropriate measures. However, while setting up policies and strategies related to HIV prevention, policymakers should consider that HIV knowledge alone does not appear to be sufficient. Further research may help to identify other factors that are essential to consider.
